# Neuronal nitric oxide synthase regulates regional brain perfusion in healthy humans

**DOI:** 10.1093/cvr/cvab155

**Published:** 2021-04-30

**Authors:** Kevin O’Gallagher, Francesca Puledda, Owen O’Daly, Matthew Ryan, Luke Dancy, Philip J Chowienczyk, Fernando Zelaya, Peter J Goadsby, Ajay M Shah

**Affiliations:** Department of Cardiology, King’s College London British Heart Foundation Centre of Research Excellence, School of Cardiovascular Medicine & Sciences, The James Black Centre, 125 Coldharbour Lane, London SE5 9NU, UK; Department of Clinical Pharmacology, King’s College London British Heart Foundation Centre of Research Excellence, School of Cardiovascular Medicine & Sciences, London, UK; Headache Group, Wolfson CARD, Institute of Psychology, Psychiatry and Neuroscience, London, UK; NIHR-Wellcome Trust King's Clinical Research Facility, SLaM Biomedical Research Centre, King's College Hospital, London, UK; Department of Neuroimaging, Centre for Neuroimaging Sciences, Institute of Psychiatry, Psychology & Neuroscience, London, UK; Department of Cardiology, King’s College London British Heart Foundation Centre of Research Excellence, School of Cardiovascular Medicine & Sciences, The James Black Centre, 125 Coldharbour Lane, London SE5 9NU, UK; Department of Cardiology, King’s College London British Heart Foundation Centre of Research Excellence, School of Cardiovascular Medicine & Sciences, The James Black Centre, 125 Coldharbour Lane, London SE5 9NU, UK; Department of Clinical Pharmacology, King’s College London British Heart Foundation Centre of Research Excellence, School of Cardiovascular Medicine & Sciences, London, UK; Department of Neuroimaging, Centre for Neuroimaging Sciences, Institute of Psychiatry, Psychology & Neuroscience, London, UK; Headache Group, Wolfson CARD, Institute of Psychology, Psychiatry and Neuroscience, London, UK; NIHR-Wellcome Trust King's Clinical Research Facility, SLaM Biomedical Research Centre, King's College Hospital, London, UK; Department of Cardiology, King’s College London British Heart Foundation Centre of Research Excellence, School of Cardiovascular Medicine & Sciences, The James Black Centre, 125 Coldharbour Lane, London SE5 9NU, UK

**Keywords:** Neuronal nitric oxide synthase, Cerebral blood flow, Vascular, Brain

## Abstract

**Aims:**

Neuronal nitric oxide synthase (nNOS) is highly expressed within the cardiovascular and nervous systems. Studies in genetically modified mice suggest roles in brain blood flow regulation while dysfunctional nNOS signalling is implicated in cerebrovascular ischaemia and migraine. Previous human studies have investigated the effects of non-selective NOS inhibition but there has been no direct investigation of the role of nNOS in human cerebrovascular regulation. We hypothesized that inhibition of the tonic effects of nNOS would result in global or localized changes in cerebral blood flow (CBF), as well as changes in functional brain connectivity.

**Methods and results:**

We investigated the acute effects of a selective nNOS inhibitor, S-methyl-L-thiocitrulline (SMTC), on CBF and brain functional connectivity in healthy human volunteers (*n *=* *19). We performed a randomized, placebo-controlled, crossover study with either intravenous SMTC or placebo, using magnetic resonance imaging protocols with arterial spin labelling and functional resting state neuroimaging. SMTC infusion induced an ∼4% decrease in resting global CBF [−2.3 (−0.3, −4.2) mL/100g/min, mean (95% confidence interval, CI), *P *=* *0.02]. In a whole-brain voxel-wise factorial-design comparison of CBF maps, we identified a localized decrease in regional blood flow in the right hippocampus and parahippocampal gyrus following SMTC vs. placebo (2921 voxels; *T *=* *7.0; *x* = 36; *y* = −32; *z* = −12; *P *<* *0.001). This was accompanied by a decrease in functional connectivity to the left superior parietal lobule vs. placebo (484 voxels; *T *=* *5.02; *x* = −14; *y* = −56; *z* = 74; *P *=* *0.009). These analyses adjusted for the modest changes in mean arterial blood pressure induced by SMTC as compared to placebo [+8.7 mmHg (+1.8, +15.6), mean (95% CI), *P *=* *0.009].

**Conclusions:**

These data suggest a fundamental physiological role of nNOS in regulating regional CBF and functional connectivity in the human hippocampus. Our findings have relevance to the role of nNOS in the regulation of cerebral perfusion in health and disease.

## 1. Introduction

Nitric oxide (NO) is an important signalling molecule within the cardiovascular and nervous systems, and one of the primary molecules involved in matching local brain blood flow to neuronal activity; the phenomenon known as neurovascular coupling.^1^ Endogenous production of NO occurs via nitric oxide synthase enzymes (NOS), of which three isoforms have been described: endothelial nitric oxide synthase (eNOS), neuronal nitric oxide synthase (nNOS), and inducible nitric oxide synthase (iNOS). Both nNOS and eNOS are constitutively expressed and have tissue-specific roles within the cell types in which they were first identified and many others.nNOS is highly expressed throughout the central nervous system.^2^ Data from animal studies suggest that nNOS may have crucial physiological roles in blood flow regulation.^3^ In post-synaptic neurons, the action of nNOS is linked to the N-methyl-D-aspartate (NMDA) glutamate receptor, activation of which results in nNOS-mediated NO synthesis.^4^ NO generation has a role in neural control of the cerebral circulation,^5^ such as that mediated by the cranial parasympathetic outflow pathway.^6^ nNOS is also expressed in peripheral autonomic (or nitrergic) nerves which modulate vessel tone.^7^ When overexpressed, NO is neurotoxic, while dysfunctional nNOS signalling has been implicated in cerebrovascular disease states such as ischaemia^8^ and migraine^9^^,^^10^ as well as neurodegenerative conditions e.g. Alzheimer’s disease and Parkinson’s disease.^10^

Previous studies using an nNOS-selective inhibitor, S-methyl-L-thiocitrulline (SMTC), in humans *in vivo* demonstrated that nNOS and eNOS have distinct roles in the local regulation of vascular tone. It was demonstrated that nNOS regulates basal microvascular tone whereas eNOS is responsible for vasodilation induced by agonists (e.g. acetylcholine) or by increases in shear stress.^11^^,^^12^ Furthermore, the vasodilation in skeletal and cardiac muscle beds that occurs in response to acute mental stress appears to involve the local actions of nNOS rather than eNOS.^11^^,^^13^ More recently, through systemic administration of SMTC, it was shown that nNOS also plays a major role in the regulation of systemic vascular resistance and therefore blood pressure.^14^ Some previous studies investigated the effects of non-selective NOS inhibition on human cerebral blood flow (CBF).^15^ However, the effects of the nNOS isoform on blood flow regulation within the human brain have not been directly investigated.

We hypothesized that inhibition of the tonic effects of nNOS would result in global or localized changes in CBF, as well as changes in functional brain connectivity. To test these hypotheses, we performed a magnetic resonance imaging (MRI) placebo-controlled study to investigate the effects of SMTC administration on regional cerebral blood flow (rCBF) and on resting state functional brain connectivity in healthy humans.

## 2. Methods

### 2.1 Approvals

Ethical approval for the study was obtained from the London Queen Square Research Ethics Committee: 18/LO/0732. Written informed consent was obtained from all volunteers prior to commencing any protocol-related procedures. The study complied with the *Declaration of Helsinki*.

### 2.2 Subject population and recruitment

Inclusion criteria were: healthy adults aged 18–80 years, no previous medical history, no contraindications to undergo MRI examination, no history of hypertension or headache, no intake of recreational drugs, including cannabis, in the last 12 months. We further excluded subjects who smoked more than five cigarettes or drank more than six cups of caffeinated drinks per day, or who had a history of psychosis, depression, or psychological diseases. Any participant taking recurrent medications with an action on the central nervous system was excluded from the study.

### 2.3 Study design

**Figure 1:**
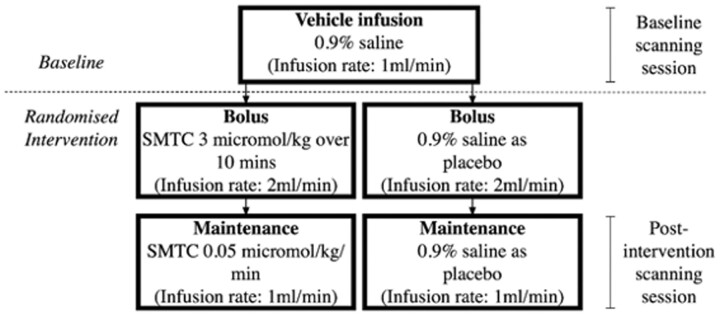
Study flow chart.

This was a randomized, placebo-controlled, crossover intervention study involving healthy volunteers. There were two visits, separated by a washout period of a minimum of one week. Each visit involved the intravenous (i.v.) infusion of either SMTC or placebo: a 10-min bolus followed by a maintenance infusion. The order of the intervention across the two visits was assigned randomly for each subject, determined by randomization software. Brain imaging at each visit involved a baseline session, followed immediately by the randomized intervention bolus, then recommencement of scanning with the maintenance infusion running (see *Figure 1* for flowchart). This was repeated in an identical fashion at each visit. Participants were blinded to the intervention in a single-blind design.

### 2.4 Infusion protocol

First, an i.v. infusion of 0.9% saline solution was given at a rate of 1 mL/min during the baseline scanning period, followed by a bolus and maintenance i.v. infusion of either SMTC or 0.9% saline. The SMTC infusion consisted of a bolus of 3.0 µmol/kg over 10 min at a rate of 2 mL/min, followed by a maintenance infusion of 0.05 µmol/kg/min for the duration of the second scanning period, administered at a rate of 1 mL/min. For a 10-min bolus followed by a 40-min infusion, the total dose of SMTC administered was approximately 5 µmol/kg. This dosing regime has been validated and shown to be nNOS-selective and safe.^14^ The i.v. infusion of 0.9% saline as placebo was given at the same infusion rate as the SMTC bolus and maintenance infusions.

Systolic blood pressure (SBP), diastolic blood pressure (DBP), and heart rate were measured before, during and following the end of the infusion. Mean arterial pressure (MAP) was derived from SBP and DBP measures.

All visits took place at the same time of day, between 9 and 12 a.m., to control for diurnal variation in regional and global brain perfusion.^16^ Subjects were instructed to consume a light breakfast and avoid caffeine from midnight on the morning of the study visit. Participants were also asked to refrain from the use of alcohol or any medication for 24 h prior to the visit. If this was not avoidable, the visit was postponed.

### 2.5 Endpoints

The primary endpoint was the effect of SMTC on rCBF. An additional, exploratory, endpoint was the effect of SMTC on resting functional brain connectivity of regions exhibiting perfusion changes.

### 2.6 Scanning procedure

All scans were conducted on a 3T General Electric MR750 MRI scanner using a 12-channel receive-only head coil at the Clinical Research Facility, King’s College Hospital, London, UK. In each study visit, scanning was conducted over two sessions, before and after the intervention. The two sets of scans were identical, except for the acquisition of structural images, which was not repeated after the intervention.

For each session, two CBF maps were generated by means of a 3D pseudo-continuous Arterial Spin Labelling (3D-pCASL) protocol.^17^ This was followed by one resting state BOLD (Blood Oxygen Level Dependent) functional magnetic resonance (rsfMRI) echo-planar image acquisition to determine changes in resting functional connectivity across the entire brain volume. Total scanning time was 6 min and 20 s for each pCASL scan and 8 min for rsfMRI.

### 2.7 Imaging parameters

High resolution 3D T1-weighted IR-SPGR images had the following characteristics: TR = 7.312 ms; TE = 3.016 ms; TI = 400 ms; flip angle = 11°; FOV = 270 mm; matrix = 256 × 256; slice thickness = 1.2 mm; 196 slice partitions, ASSET factor = 1.75.^18^

For whole brain CBF maps, labelling of arterial blood was achieved with a 1825 ms train of Hanning shaped RF pulses of 500 µs duration in the presence of a net magnetic field gradient along the flow direction (the *z*-axis of the magnet). After a post-labelling delay of 2025 ms, a whole brain volume was read using a 3D inter-leaved ‘stack-of-spirals’ Fast Spin Echo readout, consisting of 8 interleaved spiral arms in the in-plane direction, with 512 points per spiral interleave. The images had 60 axial slice locations (3 mm thickness) and an in-plane FOV of 240 × 240 mm after transformation to a rectangular matrix (TE/TR = 11.088/5180 ms, flip angle = 111°). A proton density image volume with the same parameters was acquired within the same sequence to use as a reference to compute the CBF maps in conventional physiological units (mL blood/100 g tissue/min). The sequence used four background suppression pulses to minimize static tissue signal at the time of image acquisition. Four control-label pairs were acquired. CBF maps were computed from the mean perfusion weighted difference image derived from the four control-label pairs, by scaling the difference image against a proton density image acquired at the end of the sequence, using identical readout parameters.

Multi-echo echo-planar imaging (ME-EPI) images for rsfMRI were acquired while subjects were at rest with their eyes open and had the following characteristics: TR = 2500 ms; echo times = 12, 28, 44, 60 ms; flip angle= 80°; FOV = 240 mm; matrix = 64 × 64; 32 axial sections collected with sequential (top down) acquisition and 1 mm interslice gap. One hundred and ninety-two whole brain volumes were acquired in each time series.

### 2.8 Preprocessing and analysis of pCASL data

pCASL images were preprocessed using Automated Software for ASL Processing (ASAP^19^) which employs functions within the Statistical Parametric Mapping software suite, version 12 (SPM 12; www.fil.ion.ucl.ac.uk/spm/) in MATLAB R2017a (https://uk.mathworks.com/). Voxel-wise computation of CBF was performed by the scanner software, using the formula recommended by the recent ASL consensus article^20^:

$$\mathrm{CBF}=6000\frac{e^{w/T_{1a}}}{2\varepsilon T_{1a}(\left.1-e^{-\tau /T_{1a}}\right)}\frac{P}{\frac{R}{\lambda }}$$

in which P is the signal in the mean perfusion-weighted image (from the four control-label pairs), R is the signal in the reference image, ε is the combined efficiency of labelling and background suppression (∼65%), w the postlabelling delay (2025 ms), τ is the label duration (1825 ms), $T_{1a}$ is the $T_{1}$ of arterial water, and w is the postlabelling delay (2025 ms).

For spatial normalization of the CBF maps to the space of the Montreal Neurological Institute (MNI) within the ASAP framework, a multistep approach was used: CBF maps were co-registered to the high-resolution T1-weighted structural ADNI images, after coarse alignment of the origin of both images. Unified segmentation of the T1-weighted image normalized this image to the MNI space and was used to produce a ‘brain-only’ binary mask which was multiplied by the co-registered rCBF map to produce an image free of extra-cerebral artefacts. The spatial affine and non-linear transformations were applied to the clean CBF images and then the images were smoothed using an 8 × 8 × 8 mm Gaussian kernel as described.^19^

### 2.9 rsfMRI processing and analysis

After resetting of the origins for both T1-weighted and ME-EP images, the 60 ms echo was discarded due to low signal. The remaining three echoes were taken forward to pre-processing as described below. The ME-EPI echoes were separated into distinct time series (corresponding to the three remaining individual echoes), which were then de-spiked using 3dDespike in the Analysis of Functional NeuroImages (AFNI) framework (https://afni.nimh.nih.gov), and slice time corrected. Parameters for motion correction were estimated for the first echo, and subsequently applied to the other echoes; all ME-EP images were then co-registered to the T1 scan. All echoes were spatially normalized to the study-specific template, and from there to MNI space. Finally, the images from all echoes were z-concatenated for further processing, i.e. the space-by-time matrices from each echo were appended to one another in the z-direction to form a single matrix using the *3dZcat* function in AFNI. TEDANA, a python script that forms part of the Multi Echo Independent Component Analysis (MEICA) package (https://afni.nimh.nih.gov/pub/dist/src/pkundu/meica.py)^21^^,^^22^ was called to perform TE dependent ICA-based denoizing and T2* weighted averaging (optimal combination) of echoes. The denoized, optimally combined images were subsequently regressed out for motion correction, white matter signal and cerebrospinal fluid signal. Band-pass-filtering was applied with AFNI (frequency range 0.08–0.01 Hz).

For the resting state analysis, we defined the seed of interest based on the results of the pCASL analysis, and investigated functional connectivity changes from this region to the whole brain.

### 2.10 Voxel-wise factorial design

For all pCASL and resting state fMRI images, whole brain, voxel-wise analyses were performed using a general linear model in SPM 12. Significance was defined with an initial cluster-forming voxel threshold of *P *<* *0.001 and afterwards, family wise error (FWE) correction, on the basis of cluster extent was employed, using *P *<* *0.05 as the threshold for significance, using the Gaussian random field theory.^23^

A whole brain voxel-wise flexible-factorial design using two-way analysis of variance (ANOVA) allowed analysis of changes, respectively, in regional CBF and resting state connectivity related to both visit (placebo vs. SMTC) and condition (before vs. after infusion) effects. The following variables: mean arterial blood pressure, age, gender, and handedness were added as covariates in our model for all analyses.

All brain locations are reported as *x, y*, and *z* coordinates in Montreal Neurologic Institute space; cluster size is reported as k. A neuroanatomy atlas^24^ as well as the Harvard-Oxford cortical and subcortical structural atlases from the FSL software (FSL 5; https://fsl.fmrib.ox.ac.uk/fsl/fslwiki) were used to identify the correct anatomical locations of clusters of statistically significant changes within MNI space.

For pCASL scans only, we measured the global grey matter blood flow signal using the ASAP toolbox,^19^ by extracting the average CBF values from a grey matter mask of each subject. Probabilistic grey matter images in MNI space, derived from the FSL voxel-based morphometry toolbox, were thresholded to produce a mask which included all voxels with a >20% likelihood of being grey matter. Mean global CBF for all scans before and after the infusions, with either SMTC or placebo, were compared with paired *t*-test.

### 2.11 Sample size

Our power calculations were based on previous investigations of pharmacological effects on CBF,^25^^,^^26^ studies of the sensitivity of ASL,^25^ and our own power calculations using ‘G*Power’.^27^ We computed the number of subjects needed to determine a 5% change in CBF (from a mean of 55 mL blood/100 g tissue/min) and a standard deviation of 10%. Assuming a correlation between groups of 0.8, the calculations suggested that we required 17 subjects to determine a statistically significant change in a two-tailed, paired *t*-test. To allow for ∼10% drop-out, we recruited 19 subjects.

Careful examination of similar investigations of pharmacological effects on regional functional connectivity, showed that this number of subjects (*n *=* *17–20) would also be adequate to assess the effects of SMTC on resting functional connectivity.

### 2.12 Statistical analysis of non-imaging data

Demographic and haemodynamic data were analysed with GraphPad Prism 8.0 (GraphPad Software Inc.). Data were assessed for normality using the Shapiro–Wilk test. Demographic data are presented as mean ± standard deviation, experimental data are expressed as mean ± standard error of the mean (SEM) unless otherwise stated [e.g. non-parametric statistics (median and interquartile range) for non-normally distributed data]. Blood pressure and heart rate were compared by ANOVA with repeated measures testing.

## 3. Results

A total of 19 healthy subjects participated in the study, each making two visits. None were taking regular medications or had a significant past medical history. The demographic characteristics of participants are shown in *Table 1*. There were no significant differences in baseline blood pressure or heart rate between the two study visits. CONSORT checklist and flow diagram are included in Supplementary material online, *Table S1* and *Figure S1*.

**Table 1:** Baseline characteristics of participants

Age (years)	21 (20–25)
Male gender (%)	*n *=* *10 (52.6)
Right handedness (%)	*n *=* *16 (84.2)
Haemoglobin (g/dL)	140.1 ± 17.1
Creatinine (µmol/L)	74.2 ± 11.2
Heart rate (b.p.m.)	67.0 ± 11.7
Systolic blood pressure (mmHg)	110.3 ± 12.4
Diastolic blood pressure (mmHg)	65.9 ± 9.8
Mean arterial blood pressure (mmHg)	80.7 ± 10.1

Data expressed as mean ± SD, apart from age which is expressed as median (interquartile range).

### 3.1 Peripheral haemodynamic response to SMTC infusion

**Figure 2:**
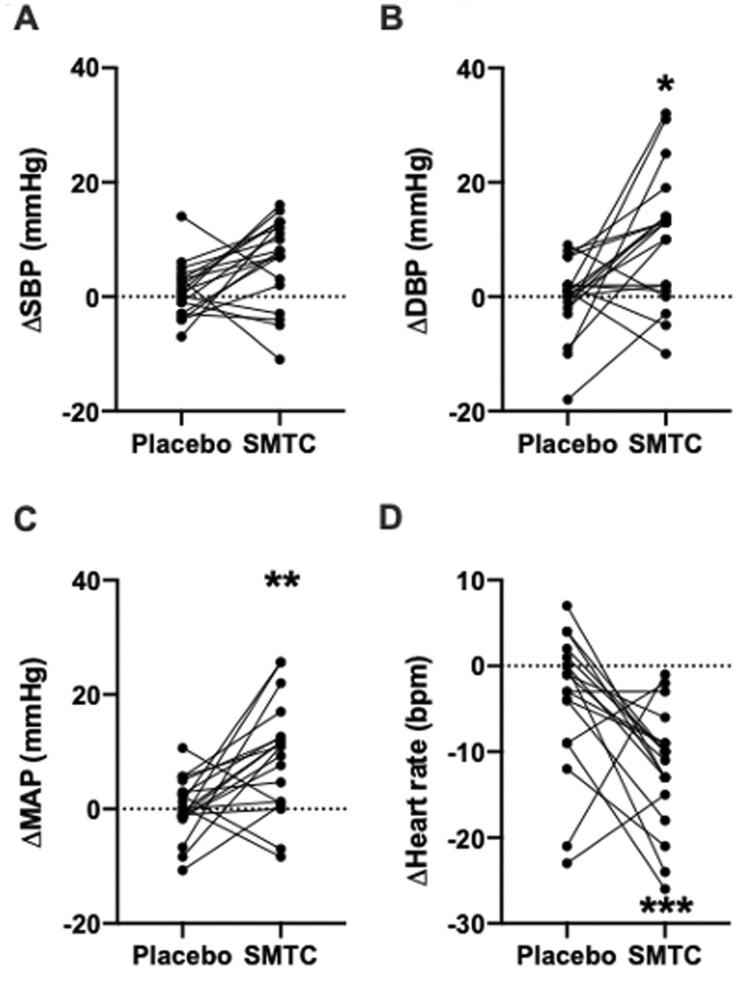
Haemodynamic effects of SMTC, represented as change from baseline. (*A*) Systolic blood pressure, SBP; (*B*) diastolic blood pressure, DBP; (*C*) mean arterial pressure, MAP; and (*D*) heart rate. *P <* *0.05, **P <* *0.01, ***P <* *0.001 as analysed by two-way ANOVA. *n* = 19.

There was no significant change in SBP following SMTC infusion as compared to placebo: +5.4 mmHg (−0.2, +11.0) [mean (95% confidence interval, CI)], *P *=* *0.06, *Figure 2A*. There was a significant increase in DBP [+10.4 mmHg (+1.5, +19.2), *P *=* *0.02, *Figure 2B*] and MAP [+8.7 mmHg (+1.8, +15.6), *P *=* *0.009, *Figure 2C*] with SMTC compared to placebo. Heart rate decreased significantly following SMTC vs. placebo [−7.9 b.p.m. (−12.8, −3), *P *=* *0.0003, *Figure 2D*]. Individual data for heart rate and blood pressure are shown in Supplementary material online, *Figure S2*. Blood pressure returned to normal after cessation of infusion in all subjects.

### 3.2 Cerebral blood flow analysis

**Figure 3:**
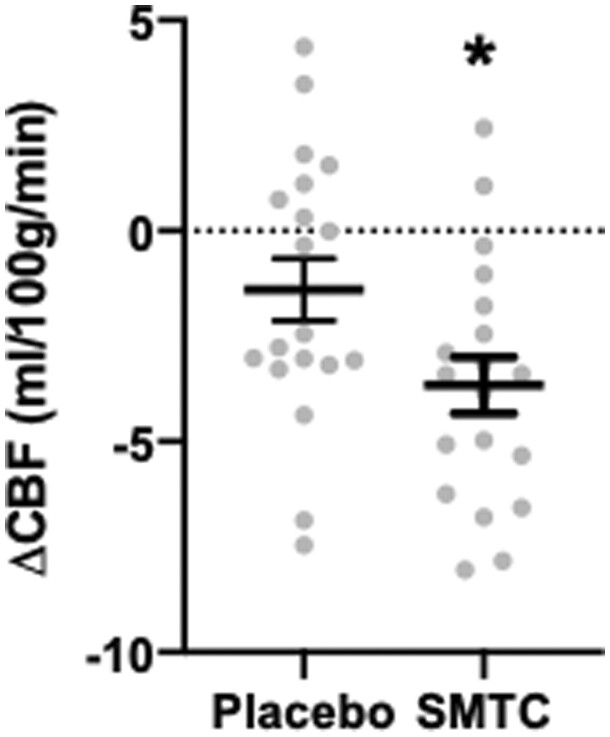
Effect of SMTC on change in global cerebral blood flow (pre- vs. post-infusion) as analysed by paired t test. *P <* *0.05. *n* = 19.

Mean global CBF was significantly decreased by ∼4% following SMTC infusion compared to placebo: −2.3 mL/100g/min (−0.3, −4.2) [mean (95% CI), *P *=* *0.02, *Figure 3*]. The mean global CBF before infusion was 55.1 ± 1.7 mL/100g/min (mean ± SEM) in the placebo arm and 56.1 ± 1.9 mL/100g/min in the SMTC arm. Post-infusion CBF was 53.7 ± 2.0 mL/100g/min in the placebo arm and 52.4 ± 1.8 mL/100 g/min in the SMTC arm.

**Figure 4:**
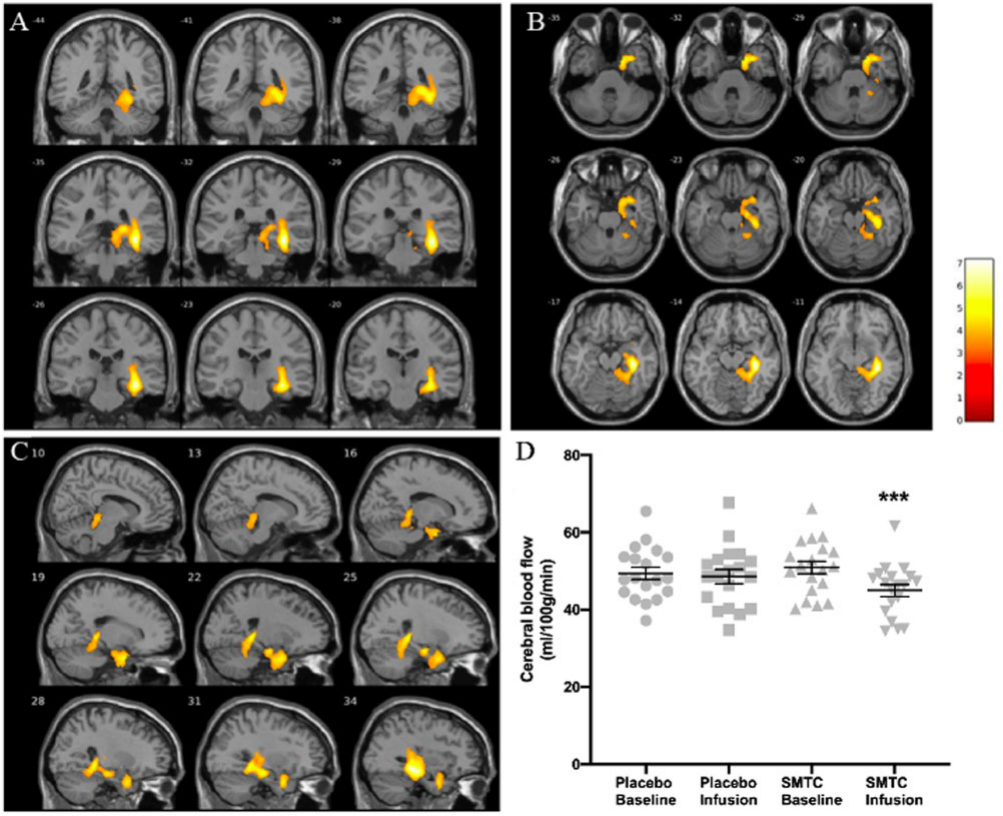
Decreased cerebral blood flow in right hippocampus and parahippocampus following SMTC infusion compared to placebo, when testing for visit and condition interaction and covarying for age, gender, handedness, and changes in mean arterial pressure (*n* = 19). Coronal (*A*), axial (*B*), and sagittal (*C*) views of the cluster are shown, as well as individual data points (with mean ± SEM) for CBF values for the cluster in each condition (*D*). Bar represents *T* values. Number of voxels = 2921; MNI coordinates: *x* = 36, *y* = −32, *z* = −12; ***P <* *0.001 as analysed by two-way ANOVA.

A whole brain voxel-wise ANOVA comparison of the CBF maps for all subjects, with testing for the interaction between type of visit (placebo vs. SMTC) and condition (before and after infusion), revealed a large cluster of localized decrease in regional CBF in the right hippocampus, parahippocampal gyrus and medial temporal lobe after SMTC infusion (number of voxels = 2921; *T *=* *7.0; *x* = 36, *y* = −32, *z* = −12; *P *<* *0.001 FWE corrected; *Figure 4*). This effect was driven by a much higher localized reduction in CBF from baseline following SMTC, compared to that seen following the placebo infusion (*Figure 4D*). We did not observe a similar effect in the contralateral hemisphere, even when using a more lenient uncorrected threshold of *P *<* *0.01. We found no significant influence of participant habituation to the scanning procedure on change in rCBF, see Supplementary material online, *Figure S3*.

### 3.3 Resting state connectivity analysis

For the resting state analysis, our seed region of interest was defined using the cluster showing reduced rCBF following SMTC infusion with respect to placebo (see above for full details of this cluster). Following Fisher’s r-to-Z transformation of connectivity r-maps, we tested for an interaction between type of visit (placebo vs. SMTC) and condition (before and after infusion) on whole-brain connectivity for this seed. One subject did not complete the resting state acquisition due to time constraints on the day of the scanning; the final analysis for this exploratory endpoint was therefore performed on 18 subjects.

**Figure 5:**
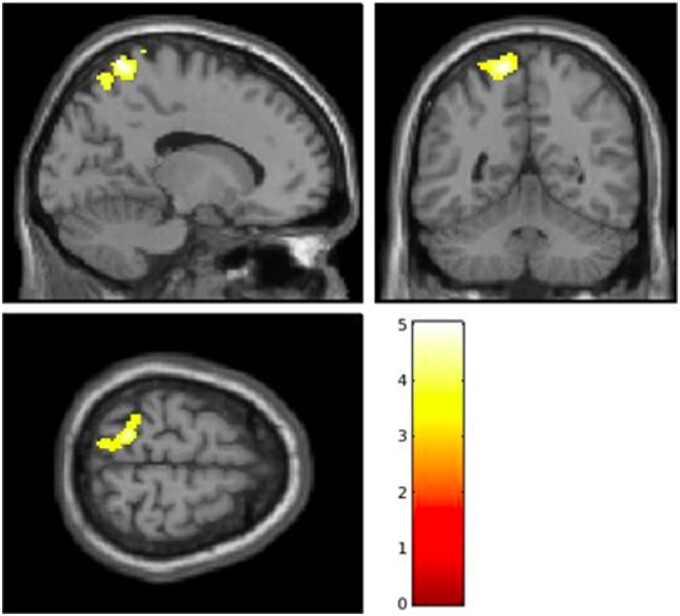
Decreased functional connectivity between the right hippocampal formation (region shown in *Figure 2*) and the left superior parietal lobule (shown here), following SMTC infusion compared to placebo (number of voxels = 484; *T *=* *5.02; *x* = −14; *y* = −56; *z* = 74; *P *=* *0.009, as analysed by two-way ANOVA) when testing for visit and condition interaction and covarying for age, gender, handedness, and changes in mean arterial pressure (*n* = 18). Bar represents *T* values.

The analysis revealed a hypoconnectivity, following the infusion and comparing SMTC to placebo (and using MAP as a co-variate), between the region showing a significant rCBF change (encompassing the hippocampus, parahippocampal gyrus and medial temporal lobe) and the left superior parietal lobule (SPL, number of voxels = 484; *T *=* *5.02; *x* = −14, *y* = −56, *z* = 74; *P *=* *0.009; *Figure 5*) during the resting state.

### 3.4 Safety

No significant adverse events were encountered. Specifically, there were no adverse behavioural or cognitive changes described by subjects following exposure to SMTC. No subject had significant hypo- or hypertension during the study. Three volunteers described minor discomfort in the infused arm during the SMTC bolus infusion; this effect was self-limiting and was not present during the maintenance infusion.

## 4. Discussion

This is the first *in vivo* human study to investigate the role of the nNOS isoform in the regulation of rCBF. We found that inhibition of nNOS through administration of SMTC results in a small, significant decrease in global CBF, with a more marked decrease in rCBF within the hippocampus and parahippocampus, areas of the brain in which nNOS is highly expressed.^28^ These changes were matched by a disruption in functional connectivity between this regional cluster and other key brain areas. The data are consistent with results from earlier animal work on the study of cerebral nitrergic mechanisms and suggest a fundamental physiological role for nNOS in regulating rCBF and brain activity.

### 4.1 Changes in regional cerebral blood flow following nNOS inhibition

nNOS-mediated influences on CBF may involve a change in cerebral conduit artery calibre and/or changes in arteriolar and capillary (i.e. microvascular) resistance. In other vascular beds such as the forearm or coronary arterial bed, SMTC has a large effect on basal coronary blood flow (up to 30% or more) but a relatively minor effect on conduit artery diameter (<4%),^11^^,^^12^ suggesting that the major effect of nNOS-derived NO may be on the cerebral microvasculature rather than on the conduit vessels. Systemic changes in vascular resistance and blood pressure, on the other hand, are unlikely to play a large role given that the effect of SMTC on MAP is within the range of cerebral autoregulation. Furthermore, the reduction in regional CBF remained significant in models that included altered MAP as a covariate.

An effect on the cerebral microvasculature would be consistent with a role for nNOS in the regulation of neurovascular coupling whereby local blood flow is matched to neuronal activity to ensure that blood supply meets metabolic demand. This linkage is facilitated by the close anatomical relationship of the cerebral arterioles to neurons and astrocytes (allowing diffusion of NO from neuron to arteriole), and also by the co-localization of nNOS to the NDMA receptor within post-synaptic neurons. A further hint to changes in blood perfusion being driven by a neuronal effect (i.e. demand) rather than a vascular effect independent of neuronal activity (i.e. simply an increase in supply) comes from observing the effect of substances such as caffeine and acetazolamide,^29^^,^^30^ which by exerting a purely vascular mechanism cause global rather than localized changes in brain perfusion. In a prior human study that assessed the effect on visually evoked increases in CBF of intravenous infusion of a non-selective NOS inhibitor, *N*^G^‐monomethyl-l-arginine (L-NMMA), the response was inhibited by 30%.^31^ However, a study that investigated the effects of L-NMMA on increases in CBF evoked by finger movement found no effect of the NOS inhibitor.^15^ These studies are not definitive regarding the role of nNOS because L-NMMA inhibits both nNOS and eNOS. The results of the current study using SMTC suggest that nNOS may have a role in modifying neurovascular coupling in the hippocampus and medial temporal lobe. Further research is required, however, to explore whether such effects involve the actions of nNOS within the neuron/astrocyte, the cerebral arteriole, or both.

As in other arterial beds, vascular resistance throughout the cerebrovascular tree is not uniformly distributed.^32^ There is also significant heterogeneity in the structure of brain vessels and their interaction with components of the neurovascular unit as one moves from pial vessels, to penetrating arterioles and capillaries. Further research with the use of appropriate stimuli to increase CBF is therefore required to identify where in the cerebral microvascular tree nNOS-derived NO has maximal effect.

### 4.2 Pre-clinical animal data

NO has been implicated in regulating resting CBF in animal models.^33^ Preclinical rodent studies found that CBF responses to perioral stimulation in the cerebellum, as measured by laser-Doppler flowmetry, are substantially reduced (up to 90%) by the nNOS inhibitor 7-nitroindazole (7-NI).^34^ Similarly, 7-NI is capable of significantly attenuating the cortical CBF increases produced by forepaw stimulation and measured through ASL.^35^ Hypercapnic vasodilation in rat parietal cortex also involves NO production.^36^

The finding of a decreased rCBF in the hippocampus, parahippocampus and medial temporal lobe highlights a potentially selective metabolic sensitivity of these brain regions for nitrergic regulation of blood flow. This finding is supported by animal studies showing that nNOS is directly implicated in blood flow changes in the medial temporal lobe, and that this effect is mediated by glutamatergic activity.^37^ Interestingly, the rich glutamatergic transmission that characterizes medial temporal lobe regions such as the hippocampus has been thought to be the basis of their well-known vulnerability to cerebral ischaemia.^38^ It is therefore possible to hypothesize that NO might be exerting a specific regional effect in hippocampal and medial temporal lobe pathophysiology.

The strong lateralization of our results to the right hippocampus is also of interest. It is known that this brain region is characterized by structural and functional asymmetries, with unilateral specializations attributed to the left and right hemispheres.^39^^,^^40^ Specifically, the right hippocampus seems to be linked to spatial memory processing.^41^ Additional studies would be needed to investigate the specific role of SMTC on this functional dissociation.

### 4.3 Functional effects of nNOS in the brain

The changes in brain perfusion following nNOS inhibition are accompanied by a functional change within the same hippocampal and medial temporal lobe region, measured as altered neuronal connectivity to a second brain region, specifically the superior parietal lobule. The hippocampal formation and superior parietal lobule further represent important hubs within two synergistic brain networks, respectively the default mode^42^^,^^43^ and dorsal attentional^44^^,^^45^ networks, that are differentially activated when the brain is at rest (without a specific cognitive engagement) or involved in an active task.^46^ It is possible that the reduced connectivity between two key regions of these networks, as seen following SMTC infusion, could signify the importance of nitrergic mechanisms in switching between resting and active brain states. Additionally, the superior parietal lobe has a role in lateralized control of visuospatial attention and working memory—the creation of short-term representations of temporary information that no longer exists in the external environment and which is then applied to specific goal-directed behaviours.^47^^,^^48^ The disconnection between these hippocampus and superior parietal lobe could therefore underline a potential role of NO in the function of working memory or attention. As with the altered rCBF, our results showing changes in functional connectivity were highly lateralized; this might indeed be due to asymmetric functional specialization within the SPL.^49^

### 4.4 Clinical implications

Beyond a fundamental physiological role in regulating rCBF, an important question is whether nNOS-mediated signalling in humans becomes dysfunctional in disease states, as has been implicated in conditions such as cerebrovascular ischaemia, migraine and neurodegenerative diseases.^10^ Indeed, recent work has implicated nNOS dysfunction in the pathophysiology of tauopathies.^50^ The current work opens the way to new avenues of investigation of the effects of NO in regional brain activity in such conditions, as well as to nNOS as a potential therapeutic target.

In ischaemic stroke, NMDA receptor stimulation leads to increased activation of nNOS, with nNOS-derived NO leading to neurotoxicity within the ischaemic penumbra.^51^ Although nNOS is therefore a potential therapeutic target to reduce infarct size in ischaemic stroke, a strategy of direct nNOS inhibition has been considered to be potentially unfavourable given its likely key physiological functions within the nervous system and the behavioural effects seen in nNOS knockout mice.^52^ The current data, however, suggest that an acute infusion of the nNOS inhibitor SMTC is safe yet biologically active within the brain. This highlights the possibility that nNOS-inhibiting agents could be tested in cerebral ischaemia, whether through systemic infusion or via local catheter-directed delivery, e.g. at the time of mechanical thrombectomy.

Mental stress is associated with adverse cardiovascular and neurovascular outcomes.^53^ Previous work by our group has shown a role for nNOS in the local regulation of arterial blood flow in response to mental stress in both the coronary^11^ and forearm arterial beds.^13^ Furthermore, peripheral nNOS-mediated vasodilator responses to mental stress are dysfunctional in essential hypertension.^54^ In the brain, changes in rCBF in response to mental stress have been demonstrated in healthy volunteers.^55^ It would therefore be of interest to explore whether these involve nNOS and if so, whether the nNOS response is inhibited in those with hypertension and other cardiovascular diseases.

### 4.5 Limitations

We had a relatively small sample size and a single-blind study design. However, the haemodynamic effects observed with SMTC administration are consistent with those previously shown in a double-blind study.^14^ In order to maintain study feasibility, there was no vasopressor study arm to control for the small change in blood pressure seen with SMTC; however, adjusting for blood pressure in statistical analysis did not alter our findings, and the changes were well within the cerebral autoregulatory range. Study recruitment was limited to healthy volunteers and the findings are therefore not generalizable to disease populations. We did not control for partial pressures of carbon dioxide in the blood, which is an important determinant of CBF regulation at the level of the microvascular arterioles.

Although we used a validated selective nNOS inhibitor (SMTC), conclusions regarding the effects of NO are an extrapolation. While this study has demonstrated a change in functional connectivity following STMC infusion, we are unable to confirm whether that change is due to acute hypoperfusion from a decrease in rCBF or if there is a direct neuronal effect. Furthermore, it is unclear whether other factors in the complex integrated regulation of CBF would compensate in the setting of chronic nNOS inhibition.

Formal cognitive testing would have provided further information regarding the functional relevance of the findings. Indeed, such data will be important for the translational potential of nNOS inhibition and should be recorded in future studies.

In conclusion, this study provides the first direct evidence that nNOS has an important role in the regulation of regional CBF in humans. These findings, together with data suggestive of nNOS dysfunction in cerebrovascular disease states such as ischaemia and migraine (as well as neurodegenerative conditions) highlight cerebral nNOS signalling as a potential therapeutic target for further investigations in the future.

## Supplementary material

Supplementary material is available at *Cardiovascular Research* online.

## Authors’ contributions

Conception and design of study: A.M.S., P.J.G., P.J.C., F.Z., F.P., and K.O.G. Acquisition and analysis of data: K.O.G., F.P., M.R., and L.D. Randomization: K.O.G. Interpretation of data: K.O.G., F.P., F.Z., O.O.D., P.J.C., P.J.G., and A.M.S. All authors have approved the submitted version and attest to the accuracy and integrity of the work.

The authors confirm that the PI for this paper is Professor A.M. Shah and that he had direct clinical responsibility for participants.

## Funding

This work was supported by the National Institute for Health Research Biomedical Research Centre (NIHR BRC) at Guy’s & St Thomas’ NHS Foundation Trust and King’s College London (IS-BRC-1215-20006) and the NIHR SLaM Biomedical Research Centre at South London and Maudsley NHS Foundation Trust and King’s College London (IS-BRC-1215-20018), both in partnership with King’s College Hospital NHS Foundation Trust. We also acknowledge support from the British Heart Foundation (CH/1999001/11735, RE/18/2/34213 to A.M.S.); and a UK Medical Research Council Clinical Research Training Fellowship (MR/R017751/1) to K.O.G.

**Conflict of interest:** none declared.

## Supplementary Material

cvab155_Supplementary_Data

## Notes

Translational perspective Animal models have implicated dysfunctional neuronal nitric oxide synthase (nNOS)-mediated signalling in neurovascular and neurodegenerative conditions. This first study of the effects of a selective nNOS inhibitor, S-methyl-L-thiocitrulline (SMTC), on the physiological regulation of human cerebral blood flow and brain functional connectivity opens the way to investigation of the effects of nNOS in cerebrovascular disease states. We also demonstrate that an acute infusion of SMTC is safe yet biologically active within the healthy human brain. The therapeutic potential of modulating the nNOS pathway in the brain could now be investigated; e.g. whether inhibition of nNOS activity is valuable in settings of post-ischaemia excitotoxicity.
