# Supplementary material for: Neuronal nitric oxide synthase regulates regional brain perfusion in healthy humans
Source: Cardiovasc Res. 2021 Apr 30;118(5):1321–9. doi: 10.1093/cvr/cvab155 (PMC8953449; doi:10.1093/cvr/cvab155)
Supplement: cvab155_Supplementary_Data [file cvab155_supplementary_data.pdf]

Supplementary Materials

Neuronal nitric oxide synthase regulates regional brain perfusion in healthy humans

†Kevin O'Gallagher[1,4], †Francesca Puledda[2,5], Owen O'Daly[3], Matthew Ryan[1], Luke Dancy[1], Philip J Chowienczyk[4], *Fernando Zelaya[3], *Peter J Goadsby[2,5], *Ajay M Shah[1].

[1] King's College London British Heart Foundation Centre of Research Excellence, School of Cardiovascular Medicine & Sciences, Department of Cardiology,

[2] Headache Group, Wolfson CARD, Institute of Psychology, Psychiatry and Neuroscience,

[3] Centre for Neuroimaging Sciences, Department of Neuroimaging, Institute of Psychiatry, Psychology & Neuroscience,

[4] King's College London British Heart Foundation Centre of Research Excellence, School of Cardiovascular Medicine & Sciences, Department of Clinical Pharmacology,

[5] NIHR-Wellcome Trust King's Clinical Research Facility, SLaM Biomedical Research Centre, King's College Hospital, London, UK

†Joint first authors

*Joint senior authors

Supplementary Table S1. CONSORT checklist

Supplementary Figure S1. CONSORT flowchart

Supplementary Figure S2. Individual patient haemodynamic data. **A:** SBP, systolic blood pressure; **B**: DBP, diastolic blood pressure; **C**: MAP, mean arterial pressure; **D**: Heart rate. n=19. "Maintenance" denotes average of data points during maintenance infusion. **p<0.05* for SMTC vs placebo, ***p<0.01* for SMTC vs placebo as analysed by 2- way ANOVA.

Supplementary Figure S3. Individual data points for regional CBF for visit 1 and visit 2. Circles represent pre-intervention values, Squares represent post-intervention values. n=19 Error bars represent mean±SEM.

CONSORT 2010 checklist of information to include when reporting a randomised trial*

Section/Topic	Item No	Checklist item	Reported on page No
**Title and abstract**			
	1a	Identification as a randomised trial in the title	1
	1b	Structured summary of trial design, methods, results, and conclusions (for specific guidance see CONSORT for abstracts)	3
**Introduction**			
Background and objectives	2a	Scientific background and explanation of rationale	5
	2b	Specific objectives or hypotheses	6
**Methods**			
Trial design	3a	Description of trial design (such as parallel, factorial) including allocation ratio	7-8
	3b	Important changes to methods after trial commencement (such as eligibility criteria), with reasons	n/a
Participants	4a	Eligibility criteria for participants	7
	4b	Settings and locations where the data were collected	9
Interventions	5	The interventions for each group with sufficient details to allow replication, including how and when they were actually administered	8
Outcomes	6a	Completely defined pre-specified primary and secondary outcome measures, including how and when they were assessed	8-9
	6b	Any changes to trial outcomes after the trial commenced, with reasons	n/a
Sample size	7a	How sample size was determined	13
	7b	When applicable, explanation of any interim analyses and stopping guidelines	n/a
Randomisation:			
Sequence generation	8a	Method used to generate the random allocation sequence	7
	8b	Type of randomisation; details of any restriction (such as blocking and block size)	7
Allocation concealment mechanism	9	Mechanism used to implement the random allocation sequence (such as sequentially numbered containers), describing any steps taken to conceal the sequence until interventions were assigned	8
Implementation	10	Who generated the random allocation sequence, who enrolled participants, and who assigned participants to interventions	23

Blinding	11a	If done, who was blinded after assignment to interventions (for example, participants, care providers, those assessing outcomes) and how	8
	11b	If relevant, description of the similarity of interventions	8
Statistical methods	12a	Statistical methods used to compare groups for primary and secondary outcomes	13-14
	12b	Methods for additional analyses, such as subgroup analyses and adjusted analyses	n/a
**Results**			
Participant flow (a diagram is strongly recommended)	13a	For each group, the numbers of participants who were randomly assigned, received intended treatment, and were analysed for the primary outcome	14
	13b	For each group, losses and exclusions after randomisation, together with reasons	n/a
Recruitment	14a	Dates defining the periods of recruitment and follow-up	n/a
	14b	Why the trial ended or was stopped	n/a
Baseline data	15	A table showing baseline demographic and clinical characteristics for each group	38
Numbers analysed	16	For each group, number of participants (denominator) included in each analysis and whether the analysis was by original assigned groups	14
Outcomes and estimation	17a	For each primary and secondary outcome, results for each group, and the estimated effect size and its precision (such as 95% confidence interval)	14-16
	17b	For binary outcomes, presentation of both absolute and relative effect sizes is recommended	n/a
Ancillary analyses	18	Results of any other analyses performed, including subgroup analyses and adjusted analyses, distinguishing pre-specified from exploratory	n/a
Harms	19	All important harms or unintended effects in each group (for specific guidance see CONSORT for harms)	16
**Discussion**			
Limitations	20	Trial limitations, addressing sources of potential bias, imprecision, and, if relevant, multiplicity of analyses	21
Generalisability	21	Generalisability (external validity, applicability) of the trial findings	20
Interpretation	22	Interpretation consistent with results, balancing benefits and harms, and considering other relevant evidence	17
**Other information**			
Registration	23	Registration number and name of trial registry	n/a
Protocol	24	Where the full trial protocol can be accessed, if available	17-20
Funding	25	Sources of funding and other support (such as supply of drugs), role of funders	22

*We strongly recommend reading this statement in conjunction with the CONSORT 2010 Explanation and Elaboration for important clarifications on all the items. If relevant, we also recommend reading CONSORT extensions for cluster randomised trials, non-inferiority and equivalence trials, non-pharmacological treatments, herbal interventions, and pragmatic trials. Additional extensions are forthcoming: for those and for up to date references relevant to this checklist, see www.consort-statement.org.

CONSORT 2010 Flow Diagram

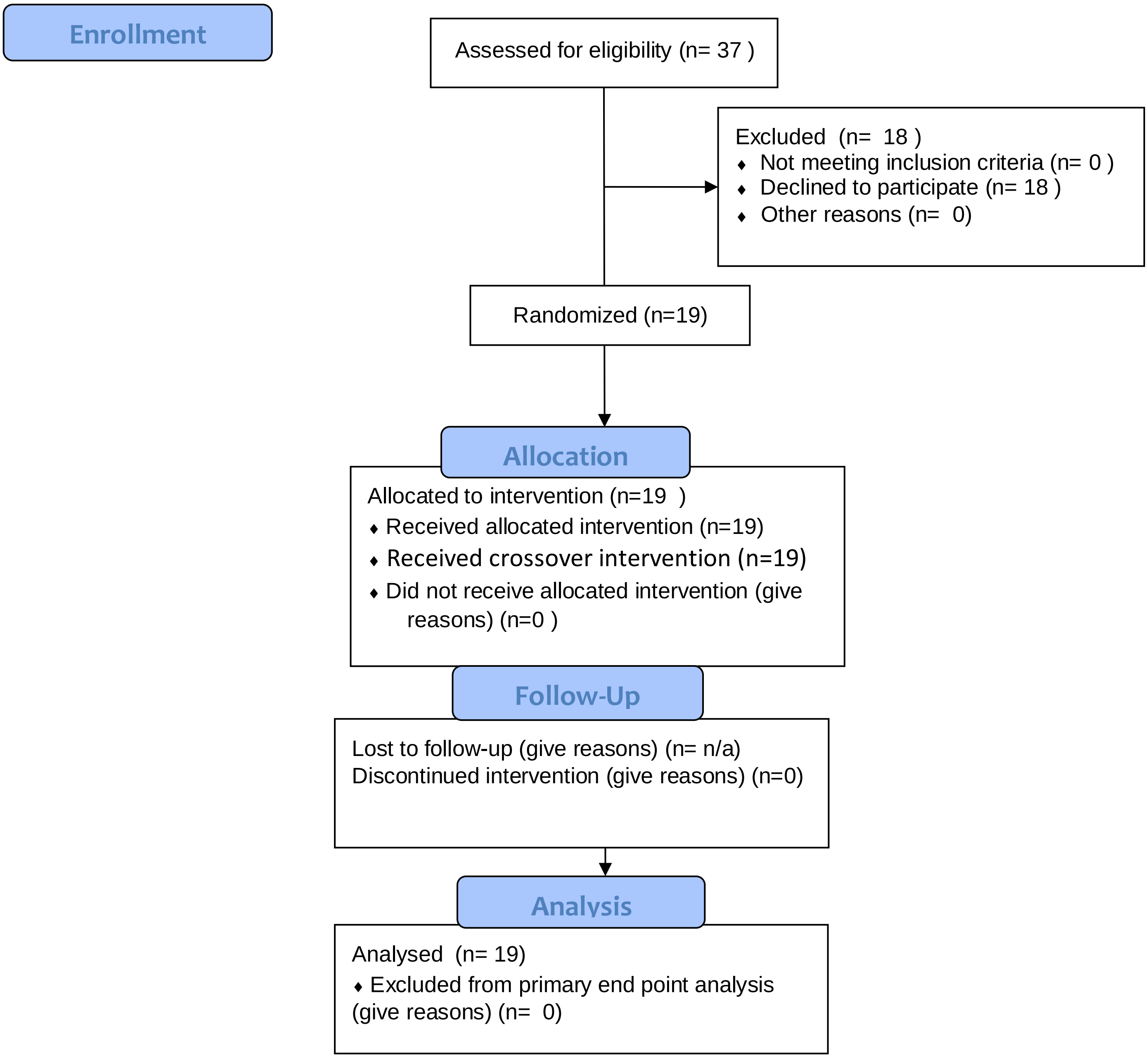

Supplementary Figure S1. CONSORT flow diagram

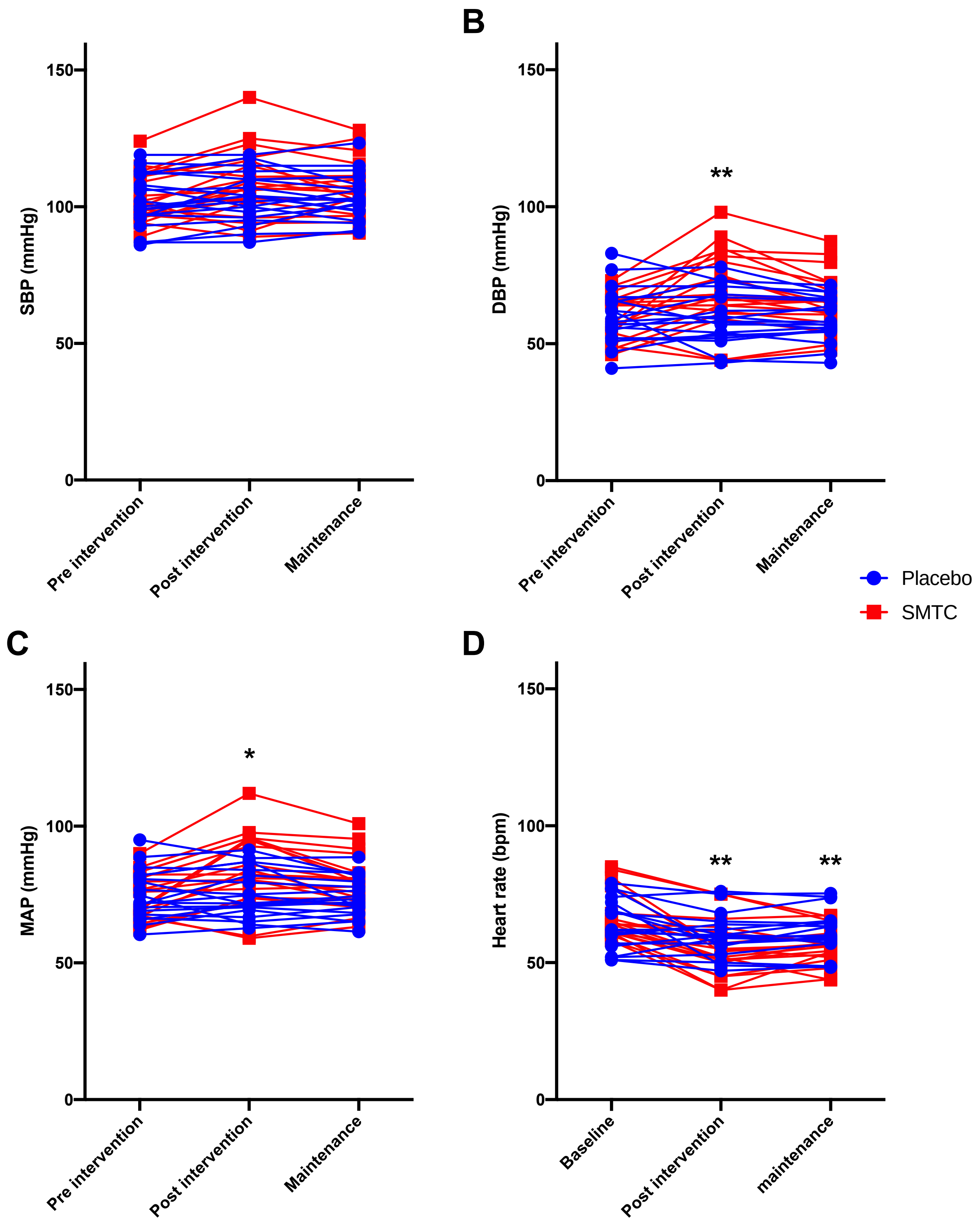

Supplementary Figure S2. Individual patient haemodynamic data. **A:** SBP, systolic blood pressure; **B**: DBP, diastolic blood pressure; **C**: MAP, mean arterial pressure; **D**: Heart rate. n=19. "Maintenance" denotes average of data points during maintenance infusion. **p<0.05* for SMTC vs placebo, ***p<0.01* for SMTC vs placebo as analysed by 2- way ANOVA.

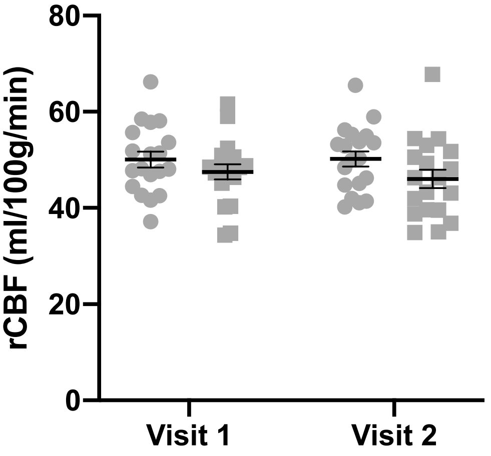

Supplementary Figure S3. Individual data points for regional CBF for visit 1 and visit 2. Circles represent pre-intervention values, Squares represent post-intervention values. n=19. Error bars represent mean±SEM.
